# Colchicine and/or Naltrexone for Hospitalized COVID-19 Patients Not Requiring High Levels of Ventilatory Support (COLTREXONE): A Prospective, Randomized, Open-Label Trial

**DOI:** 10.7759/cureus.60364

**Published:** 2024-05-15

**Authors:** Elie Gertner, Anne Schullo-Feulner, Alison Knutson, Ella Chrenka, Meghan O'Brien, Christine Behrendt, Joseph Johnson, Daniel Delaney

**Affiliations:** 1 Rheumatology, Regions Hospital, Saint Paul, USA; 2 Pharmacy, Park Nicollet Methodist Hospital, St. Louis Park, USA; 3 Statistics, HealthPartners Institute, Bloomington, USA; 4 Clinical Research, HealthPartners Institute, Saint Paul, USA; 5 Interventional Pain, HealthPartners Tria Orthopedic Center, Bloomington, USA; 6 Pharmacy, Park Nicollet Clinic, Minneapolis, USA

**Keywords:** cytokines, covid-19, naltrexone, colchicine, coltrexone

## Abstract

We assessed the efficacy and safety of colchicine and low-dose naltrexone (LDN), alone and in combination, in preventing progression to severe acute respiratory syndrome coronavirus 2 (SARS-CoV-2) infection.

In this prospective, randomized, open-label trial, colchicine and LDN were compared to standard of care (SOC) in patients hospitalized with SARS-CoV-2 not requiring high levels of ventilatory support. Patients were randomly assigned to colchicine alone, LDN alone, colchicine/LDN in combination, or SOC. The primary outcome was time to disease recovery. Secondary outcomes included total time hospitalized, study enrollment, level of care, oxygen support, and adverse events.

One-hundred and thirty-seven patients were randomized (N_c_ = 34, N_c+ldn_ = 33, N_ldn_ = 35, N_soc_ = 35). Eighty-four patients (61%) achieved disease recovery by day 5. There was no significant difference in the proportion of patients who experienced the primary efficacy outcome among those who received colchicine, LDN, or between the four study arms.Patients receiving colchicine had a shorter length of enrollment but not a significant reduction in the length of stay. Diarrhea was the most common adverse reaction.

In adults hospitalized with SARS-CoV-2 not requiring high-level ventilatory support, colchicine and LDN, alone and in combination, were not associated with significant reductions in progression to severe disease.

## Introduction

Severe acute respiratory syndrome coronavirus 2 (SARS-CoV2) infection leads to increased circulating proinflammatory chemokines and cytokines, and disease severity correlates with the level of hyperinflammatory markers [1,2]. This hyperinflammatory state also contributes to the development of acute respiratory distress syndrome (ARDS) [3], thromboembolic disease [4], and myocardial injury [5]. Therapeutic approaches that are relatively inexpensive, readily available, and target reduction of the hyperinflammatory response would be useful.

Colchicine, approved for gout and Familial Mediterranean Fever [6], is also used in acute coronary syndrome [7], pericarditis [8], and ARDS. Its use in autoimmune/autoinflammatory disorders includes Bechet’s Disease and periodic fever with aphthous stomatitis, pharyngitis, and adenitis (PFAPA). It may even be an effective treatment for children with a clinical diagnosis of autoinflammatory diseases without pathogenic gene variants [9]. Colchicine binds to tubulin, interferes with neutrophil chemotaxis, adhesion, and mobilization to sites of inflammation, and contributes to a reduction in superoxide production. Colchicine can inhibit NLRP3 inflammasome formation causing a reduction in IL-1β, IL-6, and IL-18 production [10-12]. It has an anti-apoptotic action on endothelial cells [13] which may minimize extravasation, capillary leak, and therefore development or progression of ARDS. The minimal toxicity profile and mechanism of action justified the investigation of colchicine to minimize disease progression and shorten the time to resolution of infection [14]. Colchicine in hospitalized patients has been evaluated in several comparative cohort studies, observational studies, and randomized controlled trials [15-20]. 

Naltrexone, an opioid antagonist, possesses immunomodulatory effects. Naltrexone is a 50:50 racemic mixture of both L and R isomers, which in theory boost the immune system yet limit an excessive immune response. Proposed actions of low-dose naltrexone (LDN) in the treatment of SARS-CoV-2 include its potential to act as an immune enhancer via upregulation of the endogenous opioid system, which increases endorphins and enkephalins and decreases multiple inflammatory cytokines [21]. Naltrexone may inhibit the ERK 1 / 2 pathway, which may halt viral replication [22]. The safety profile of naltrexone at standard doses (25-100 mg) is well established. Thus, 1-4.5 mg LDN daily should have little to no toxicity.

## Materials and methods

Study design

This prospective, randomized, open-label trial with a two-by-two factorial design was conducted at Regions Hospital, an academic safety net hospital, and Methodist Hospital, a community hospital, both serving high-risk, underserved, and community populations within a Midwestern healthcare system. The four treatment arms were colchicine alone, LDN alone, the combination of colchicine and LDN, and standard of care (SOC). The SOC arm was used in lieu of a blinded/placebo design to minimize healthcare worker exposure to COVID-19 and personal protective equipment consumption. CONSORT guidelines were followed. The trial was registered on ClinicalTrials.gov and assigned the identifier NCT04756128. Approval from the HealthPartners Institute Review Board, Bloomington, MN (Approval Number: A21-034) was received on January 18, 2021. Enrollment began on 25th January 2021 with the recruitment of the first participant and closed on 27th November 2021. Study data was collected from the date the first participant was enrolled and continued until the last participant was discharged from the hospital on 12th December 2021. Additional data cleaning, validation, and analysis were performed through February 2022.

Patients

**Figure 1:**
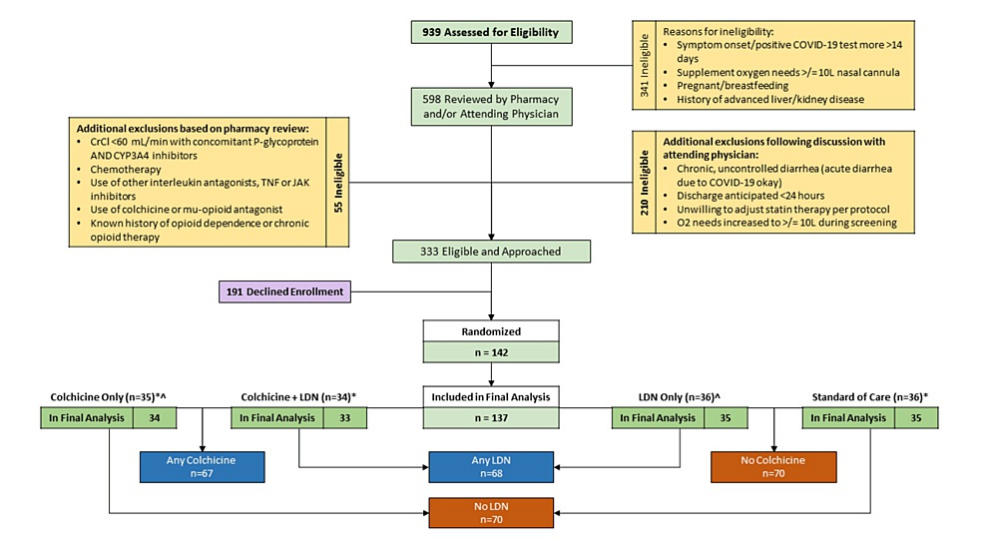
Consort diagram of study screening, enrollment, and randomization Consort diagram representing steps in the process of screening, enrolling, and randomizing COLTREXONE study participants. One-hundred and thirty-seven participants included in the final analysis were allocated to one of four arms in the following fashion: Colchicine and LDN (n=33), colchicine-only (n=45), LDN-only (n=35), and standard of care (n=35). These participants were further sorted into two additional groups as part of the 2X2 factorial study design: (a) colchicine (n=67) or no colchicine (n=70) and (b) LDN (n=68) or no LDN (n=69). *Participant withdrawn by the investigator after not meeting I/E criteria ^Participant withdrew consent for continued participation in the study LDN: Low-dose naltrexone; COVID-19: coronavirus disease 2019

Potential study candidates were identified and screened using reports of hospitalized patients with either presumed or confirmed SARS-CoV-2 infection. Eligible patients were required to (1) have a diagnosis of laboratory-confirmed COVID-19, (2) symptom onset and positive COVID-19 test both within 14 days of enrollment date, and (3) meet the criteria for moderate illness as outlined in a modified version of the World Health Organization (WHO) R&D Blueprint Ordinal Clinical Scale (Table 1). Patients with an asymptomatic/incidental COVID-19 finding, those who were pregnant or breastfeeding, and those with significant liver and kidney issues (e.g., cirrhosis, chronic kidney disease stage IV or higher) were excluded. A designated study pharmacist evaluated renal function and drug-drug interactions to ensure they were not precluded from any of the treatment arms. A consort diagram of the study’s screening, enrollment, and randomization scheme depicts key inclusion/exclusion criteria assessed at each step of the enrollment process (Figure 1). Written consent was obtained prior to the initiation of study procedures from the participant or their legally authorized representative. To provide equitable access to the study for non-English speaking patients, an abbreviated consent form translated into Spanish, Korean, Hmong, and Somali was used. This effort allowed up to 96.4% of COVID-19 patients admitted during the enrollment period to receive a consent form in their native language and decreased ineligibility due to a language barrier by 62.5%. 

**Table 1:** World Health Organization (WHO) R&D Blueprint Ordinal Clinical Scale A modified version of the World Health Organization (WHO) Research & Development Blueprint Ordinal Clinical Scale. Study eligibility required a clinical score of either 2 or 3. As study outcomes did not differentiate between those discharged with or without limitations on activities from those hospitalized but not requiring ongoing medical care (i.e., ready for discharge), they were grouped into a clinical scale score of 1 (as compared to scores 1-3 in the WHO eight-category ordinal scale) ECMO: Extracorporeal membrane oxygenation

Patients	Severity	Score	Definition
Recovered	N/A	1	Discharged or ready for discharge (i.e., not requiring further medical care or monitoring as assessed by treating physician - afebrile, blood oxygen saturations >94% on room air or baseline oxygen needs, respiratory rate < 24 per minute [all x 24 hours])
Enrolled Population	Hospitalized, moderate disease	2	Hospitalized, not requiring supplemental oxygen (but requiring ongoing medical care or monitoring). If decreasing from a score of 3, must be at baseline oxygen requirements for 24 hours.
		3	Hospitalized requiring supplemental oxygen via nasal cannula (for a minimum of 24 hours if the score increases from 2 or decreases from 4)
	Hospitalized, severe disease	4	Hospitalized on high-flow nasal cannula (HFNC) or noninvasive positive-pressure ventilation (NIPPV) support, (for a minimum of 24 hours if the score increases from 3 or decreases from 5). Patients with baseline use of NIPPV, e.g., nocturnal CPAP/BiPAP for obstructive sleep apnea must also require qualifying oxygen support during the day to obtain a score of 4.
	Hospitalized, critical disease	5	Mechanically ventilated, or a required transfer for ECMO
	N/A	6	Death

Randomization and masking

Block randomization with groups of 8 were used to ensure equal group sizes, stratified by the baseline severity score (2 or 3) to maintain balance between the four arms: (1) 0.6mg colchicine twice daily (08:00/20:00) and 4.5mg naltrexone once per day (08:00), (2) 0.6mg colchicine twice daily (08:00/20:00), (3) 4.5mg naltrexone daily (08:00), or (4) standard supportive care. Patients were considered randomized after being assigned a subject ID linked to their corresponding group. 

Procedures

Colchicine was provided as an oral tablet, and LDN as an oral suspension. The study medication was administered for up to 28 hospital days following randomization and discontinued upon hospital discharge. Patients were followed by the study team for the entire duration of their hospital stay.

All groups received standard supportive care per institutional guidelines. Such interventions included the use of respiratory support/supplemental oxygen, steroids (dexamethasone, prednisone, methylprednisolone), remdesivir, and/or tocilizumab if institutional criteria were met. Only one subject in the SOC received tocilizumab.

Patient demographics, medical history, vaccination status, and oxygen requirements were abstracted at the time of enrollment. Data points included oxygen requirements, level of care required (e.g., general medical unit, intensive care unit (ICU), and progressive/step-down ICU), clinical scale score, and adverse events. Cumulative metrics were calculated at the time of hospital discharge (e.g., length of time hospitalized, length of time requiring supplemental oxygen, doses of remdesivir received, etc.). Adherence to the treatment protocol was confirmed by a review of the participant’s list of administered medications as documented in the electronic medical record (EMR). Per standard of care, the date and time of each dose of medication were entered into a patient’s medical record by the clinical staff as it was given. This information was later collected by study staff on trial-specific data collection forms. “Missed” doses, along with the reason they were missed (e.g. participant preference, AE, staff error), were also documented.

Outcomes

The primary outcome was to determine the impact of colchicine and LDN, alone or in combination, on time to disease recovery in patients hospitalized with moderate COVID-19. Disease recovery from moderate COVID-19 was defined as achieving a clinical scale score of 1 (indicating the patient no longer required hospital-level care for COVID-19, Table 1). Patients were considered to have “moderate” COVID-19 pneumonia if they met one or more of the following criteria at screening: (1) dyspnea limiting usual activities on baseline oxygen needs, (2) respiratory rate >/= 30/min at or above baseline oxygen needs, (3) blood oxygen saturations <94% on room air (or at baseline oxygen needs if on home oxygen therapy), and (4) requiring supplemental oxygen above baseline needs. COVID-19 symptoms being ambiguous at times, attending physicians were contacted to confirm that COVID-19 was a contributing factor for the patient’s hospitalization prior to approaching patients for the study. 

The primary outcome was measured in two ways: (1) attainment of a score of 1 by Study Day 5 and (2) total post-enrollment study days required to achieve a score of 1. Attainment of a score of 1 by study day 5 was chosen based on initial experience treating COVID-19 within this specific health system; patients with similar disease severity were typically hospitalized for 6-7 days, and time from admission to enrollment in preceding COVID-19 studies was generally 1-2 days.

Those discharged after returning to their baseline oxygen needs were given a clinical scale score of 1 at the time of discharge. The care system provides a “Hospital at Home” (H@H) program for patients who require continued daily follow-up and acute management but could be safely monitored outside the hospital; these patients were assigned a clinical scale score of 1 when discharged from this service. In rare instances where the clinical score was unclear or difficult to assess, independent data abstractors compiled home-based COVID-19 documentation, redacted all identifying information, and the primary investigator performed a blinded assessment. Due to logistical constraints, those treated in the H@H program were unable to receive study medication after leaving the hospital.

Secondary outcomes examined were related to level of care and oxygen support. Days hospitalized and days of study enrollment (calculated from the time of patient’s first dose of study drug or anticipated first dose if not randomized to receive one), need for, and duration in hours of ICU/step-down ICU admission, non-invasive positive pressure ventilation (NIPPV) during enrollment, use of corticosteroids and/or remdesivir and if so, the total doses received were assessed. Other metrics of interest included the number of post-enrollment days requiring supplemental oxygen, number of post-enrollment days with a fever ≥38 degrees Celsius, and recovery from COVID-19 by study day 7 (as defined in the primary outcome).

The frequency of adverse events known to be associated with either study medication was reported for all study groups according to the following categories: (1) diarrhea/loose stools/incontinence, (2) dizziness, (3) nausea/stomach pain/vomiting, (4) anxiety/agitation, (5) headache, and (6) elevated liver function tests. Deaths were recorded as a measure of composite in-hospital mortality, and post-trial deaths were also documented.

Statistical analysis

At the time of study ideation (January 2021), the primary endpoint was defined as the proportion of patients progressing from moderate (2-3 clinical severity score) to severe/critical (4+) illness. By March 2021, this outcome was rare due to changes in care for high-risk patients leading investigators to redefine the primary endpoint. Sample size calculation was performed assuming 30% of the untreated patients would meet the new primary outcome criteria. A sample size of 136 (34/arm) was sufficient to achieve 80% power and a type I error rate of 5%, while detecting a 20% unadjusted difference between patients receiving any colchicine versus no colchicine (similarly, between patients with/out LDN). No formal power calculations were performed for comparison of individual study arms, interactions, or secondary and safety outcomes. Any discussion of these comparisons is considered unpowered and exploratory.

Due to the well-known safety and tolerability profiles of the study medications, a Data Safety and Monitoring Committee was not assigned. To avoid unnecessarily overburdening hospital staff, primary outcome data was pulled at 25% and 50% of target enrollment to assess futility. The proportion of patients achieving disease recovery by day 5 for SOC and pooled for the three intervention arms to ensure blinding by specific intervention. If an absolute difference of <10% was observed, the study would be paused, and futility and ethical considerations would be evaluated before resuming recruitment.

Demographics, clinical characteristics, and descriptors of care for the sample were described. Univariate testing was used to identify baseline differences between arms and main effect treatment groups. Variables that significantly differed between arms were used as analytic covariates. All analyses were performed based on intent-to-treat principles, defined as participants who received at least one dose of study drug. 

Outcomes were summarized overall and by study arm. For the primary outcome, chi-square analysis was performed to identify associations between treatment and the probability of achieving disease recovery by enrollment day 5. Logistic regression was used to estimate main treatment effects associated with the primary outcome. An interaction term was included to determine whether a treatment interaction effect was present. If no interaction effect (p < .05), the interaction term was excluded from final models. Adjustment for baseline clinical score along with variables that were found to be unbalanced between study arms were included in the analytic model. Analysis of secondary outcomes utilized similarly structured generalized linear models with appropriate link function. Continuous outcomes that did not meet the assumption of normality were log-transformed. Day of disease recovery was analyzed using Cox proportional hazards models including accompanying Kaplan-Meier plots. 

Sensitively analysis included adjusted regression with a set of patient covariates designated as conceptually and clinically important in predicting clinical outcome for hospitalized COVID-19 patients (sex, vaccination status, count of high-risk factors and comorbidities). To determine if there was an effect of enrollment timing within the 10-month study period, these models included an additional adjustment for a patient’s day of enrollment. Safety analysis included all participants that received at least one dose of study medication. Safety outcomes were summarized by arm and any associations with study drugs were described. All analyses were performed in SAS 9.4 (SAS Institute Inc., Cary, NC) with two-sided p-values of 0.05. 

## Results

A total of 939 patients were assessed for eligibility between January 25th, 2021, and November 29th, 2021. Of the 598 who were initially identified as eligible, 333 were ultimately approached after excluding 265 based on follow-up eligibility assessment (Figure 1). A total of 142 consented and were randomized to study arms. One hundred thirty-seven patients were included as the final analytic sample (one subject withdrew before receiving any study drug, one in mid-study, and three were found to be ineligible based on enrollment error). Randomization resulted in study arms of approximately equal size (Nc = 34, Nc+ldn = 33, Nldn = 35, Nsoc = 35). Most patients were male (n = 79, 68%), white (97, 71%), and non-Hispanic (126, 92%), with an average age of 58 (SD = 15) and BMI of 34 (SD = 9) (Table 2). At the time of enrollment, 87% (n = 116) of the patients were categorized with a clinical severity score of 3. Seventy-three percent of the sample (n = 100) were unvaccinated for COVID-19. The four arms had different representations of patients with diabetes mellitus (DM) with a higher proportion of DM patients in the colchicine arm. There was uneven representation of patients who received H@H services during their course (pc = 21%, pc+ldn = 3%, pldn = 9%, psoc = 23%). The arms were otherwise balanced (Table 2). All patients assessed for the primary endpoint received at least one dose of their specified treatment, meeting the per-protocol definition. 

**Table 2:** Patient characteristics and enrollment descriptives of the participants in the four intervention groups * p<0.05, ** p<0.01 Notes: All statistics presented as n(%) or Mean ± Standard Deviation (SD); univariate comparisons between study arms performed using Chi-Square (comparisons of proportions) and two-sample t-tests (all others); All covariates measured at the time of study enrollment except receiving hospital at home which was determined at the time of discharge; time of symptom onset patient reported; last day of enrollment – study day 304 ^a^ Count of comorbidities is summation of incidence of diabetes, hypertension, chronic renal disease, cardiovascular disease, chronic lung disease, pulmonary hypertension, chronic liver disease, and obesity ^b ^Count of high-risk factors is summation of incidence of age > 60, ejection Fraction <30%, chronic obstructive pulmonary disease (COPD), current prescription of immunosuppressive medications, and corrected QT interval (QTC) > 470 females, > 450 males ^c ^Vaccination definitions as follows: Full (1 dose of Johnson & Johnson, 2 doses mRNA), partial (1 of 2 doses of mRNA); mRNA (Pfizer or Moderna), recombinant (J&J);

Demographics	All Patients N = 137	Colchicine Only N = 34	Colchicine & LDN N = 33	LDN Only N = 35	Standard of Care N = 35
Sex - Female	58 (42%)	14 (41%)	9 (27%)	19 (54%)	16 (46%)
Age	58 ± 15	57 ± 11	55 ± 15	59 ± 16	59 ± 16
Race					
White	97 (71%)	24 (71%)	24 (3%)	24 (69%)	25 (71%)
Black or African American	19 (14%)	3 (9%)	5 (15%)	4 (11%)	7 (20%)
Hispanic	8 (6%)	3 (9%)	2 (6%)	3 (9%)	0
Asian	7 (5%)	1 (3%)	1 (3%)	3 (9%)	3 (6%)
American Indian or Alaska Native	1 (1%)	0	0	1 (3%)	0
Unknown	3 (2%)	2 (6%)	1 (3%)	0	0
Other	2 (1%)	1 (2%)	0	1 (3%)	0
Ethnicity					
Hispanic	10 (7%)	4 (12%)	3 (9%)	3 (9%)	0
Non-Hispanic	126 (92%)	29 (85%)	30 (91%)	32 (91%)	35 (100%)
Unknown	1 (1%)	1 (3%)	0	0	0
Medical Details					
BMI					
< 30	55 (40%)	13 (38%)	14 (42%)	14 (40%)	14 (40%)
30-35	23 (17%)	6 (18%)	6 (18%)	4 (11%)	7 (20%)
35-40	16 (19%)	8 (24%)	6 (18%)	8 (23%)	4(11%)
>40	33 (24%)	7 (21%)	7 (21%)	9 (26%)	10 (29%)
Number of Comorbidities ^a^					
0	21 (15%)	7 (21%)	6 (18%)	4 (11%)	4 (11%)
1	53 (39%)	11 (32%)	10 (30%)	14 (40%)	18 (51%)
2	36 (26%)	4 (12%)	11 (33%)	11 (31%)	10 (29%)
3	17 (12%)	8 (24%)	4 (12%)	4 (11%)	1 (3%)
4	8 (6%)	3 (9%)	2 (6%)	2 (6%)	1 (3%)
5	2 (1%)	1 (3%)	0	0	1 (3%)
Diabetes**	39 (28%)	17 (50%)	9 (27%)	9 (26%)	4 (11%)
Hypertension	69 (50%)	15 (44%)	14 (42%)	20 (57%)	20 (57%)
Chronic Renal Disease	9 (7%)	1 (3%)	3 (9%)	3 (9%)	2 (6%)
Cardiovascular Disease	18 (13%)	4 (12%)	5 (15%)	3 (9%)	6 (17%)
Chronic Lung Disease	18 (13%)	5 (15%)	5 (15%)	5 (14%)	3 (9%)
Pulmonary Hypertension	2 (1%)	0	0	1 (3%)	1 (3%)
Chronic Liver Disease	6 (4%)	3 (9%)	2 (6%)	1 (3%)	0
Obesity	57 (42%)	15 (44%)	14 (42%)	14 (40%)	14 (40%)
Number of Risk Factors ^b^					
0	66 (48%)	17 (50%)	18 (55%)	13 (37%)	18 (51%)
1	48 (35%)	12 (35%)	11 (33%)	14 (40%)	11 (31%)
2	19 (14%)	4 (12%)	4 (12%)	6 (17%)	5 (14%)
3	4 (3%)	1 (3%)	0	2 (6%)	1 (3%)
Age > 60	57 (42%)	14 (41%)	12 (36%)	18 (51%)	13 (37%)
EF < 30%	2 (1%)	0	0	1 (3%)	1 (3%)
Moderate/Severe COPD	6 (4%)	2 (6%)	0	3 (9%)	1 (3%)
Immunosuppressive Medications	4 (3%)	0	1 (3%)	2 (6%)	1 (3%)
QTC > 470 F, > 450 M	29 (21%)	7 (21%)	6 (18%)	8 (23%)	8 (23%)
Vaccination Status					
No Vaccination	100	25	26	22	27
At Least One Dose of Vaccine ^c^	37 (27%)	9 (26%)	7 (21%)	13 (37%)	8 (23%)
Full: mRNA	20	5	3	9	3
Full: Recombinant	4	1	1	1	1
Partial	11	3	2	3	3
Unknown	2	-	1	-	1
Enrollment and Care					
Time From the Symptom Onset Prior to Presentation at the Hospital					
0 - 7 Days	70 (51%)	16 (47%)	19 (58%)	16 (46%)	19 (54%)
8 - 14 Days	66 (48%)	18 (53%)	14 (42%)	18 (51%)	16 (46%)
Unknown	1 (1%)	0	0	1 (3%)	0
Days between Admission and Enrollment	1.1 ± 0.7	1.1 ± 0.7	1.0 ± 0.7	1.2 ± 0.7	1.1 ± 0.6
Baseline Clinical Scale Score					
2	18 (13%)	4 (12%)	5 (15%)	4 (11%)	5 (14%)
3	119 (87%)	30 (88%)	28 (85%)	31 (89%)	30 (86%)
Oxygen Needs at the Time of Enrollment					
Room Air	20 (15%)	4 (12%)	6 (18%)	5 (14%)	5 (14%)
1-3L	61 (45%)	12 (25%)	15 (45%)	16 (465)	18 (51%)
4-6L	41 (30%)	12 (35%)	9 (27%)	11 (32%)	9(26%)
7-9L	15 (11%)	6 (18%)	3 (9%)	3 (9%)	3 (9%)
Day of Enrollment	150 ± 91	147 ± 89	156 ± 96	153 ± 94	145 ± 90
Received Hospital at Home*	19 (14%)	7 (21%)	1 (3%)	3 (9%)	8 (23%)

Of the 137 study patients, 61% (n = 84) achieved disease recovery by study day 5. There was no significant difference in the proportion of patients achieving disease recovery by enrollment day 5 associated with colchicine (69% versus 54%, χ2= 2.9, df = 1, p=0.08), LDN (65% versus 58%, χ2 = 0.7, df = 1, p=0.42), or between the four study arms (χ2= 3.7, df = 3, p=0.30) (Table 3). Preliminary models did not find a significant interaction effect between treatments (p=0.84). Therefore, the primary analytic model only included the two main effects, a priori determined adjustment for baseline clinical severity score and the variables not balanced between study arms (i.e., diabetes diagnosis and H@H services). The main analytic models did not find a significant treatment effect of Colchicine (aOR 1.76; 95% CI: 0.83-3.72, p=.14) or LDN (aOR; 1.04; 95% CI: 0.49-2.19, p=.92) (Table 4). Patients who received H@H care were 81% less likely to achieve disease recovery by day 5 of study enrollment (95% CI: 0.06-0.58, p<.01). 

**Table 3:** Comparisons of primary and secondary outcomes by the treatment group and study arm * p<0.05, ** p<0.01 IQR: Interquartile range; HFNC: high-flow nasal cannula; NIPPV: non-invasive positive pressure ventilation; ICU: intensive care unit Notes: All statistics presented as n(%) or median (IQR); univariate comparisons between study arms performed using Chi-Square (comparisons of proportions) and Wilcoxon tests (all others); length of stay/enrollment defined as number of days from admission/enrollment to discontinuation of care (discharge from the hospital or last day of hospital at home); day of disease recovery, cumulative remdesivir (doses) and steroids (mg) comparisons only using patients who met/required outcome (disease recovery: n = 134, remdesivir: n = 92, steroids: n = 129).

Outcome	All Patients	By Study Arm				Treatment Group Comparisons	
		Colchicine Only	LDN & Colchicine	LDN Only	Standard of Care	Colchicine vs No Colchicine	LDN vs No LDN
N	137	34	33	35	35	67 vs 70	68 vs 69
Achieved Disease Recovery by Day 5	84 (61%)	22 (65%)	24 (73%)	20 (57%)	18 (51%)	69% vs 54%	65% vs 58%
Achieved Disease Recovery	134 (98%)	34 (100%)	33 (100%)	32 (91%)	35 (100%)	100% vs 96%	95% vs 100%
Day of Disease Recovery	5 (3-8)	4.5 (3-8)	5 (4-6)	5 (3-8)	5 (4-9)	5 (3-6) vs 5 (4-9)	5 (3-7) vs 5 (4-9)
Length of Stay, days	4.9 (4-9)	4.5 (3-8)	4.7 (4-6)	5.7 (3-10)	6.0 (5-10)	4.7 (4-7) vs 5.7 (4-10)	4.8 (3-9) vs 4.9 (4-10)
Length of Enrollment, days	3.9 (2-7)	3.5 (2-7)	3.7 (3-5)	4.6 (2-8)	4.3 (3-8)	3.7 (2-5) vs 4.5 (3-8)*	3.8 (2-7) vs 3.9 (3-8)
Received Remdesivir	92 (67%)	20 (59%)	23 (70%)	25 (71%)	24 (69%)	64% vs 70%	71% vs 64%
Remdesivir Amount (doses)	5 (3-5)	4.5 (3-5)	4 (2-5)	5 (3-5)	5 (4-5)	4 (3-5) vs 5 (4-5)	4 (3-5) vs 5 (3-5)
Received Steroids	129 (94%)	31 (91%)	31 (94%)	34 (97%)	33 (94%)	93% vs 96%	96% vs 93%
Steroid Amount (mg)	213 (160-356)	200 (160-360)	200 (147-240)	240 (120-360)	240 (200-400)	200 (160-280) vs 240 (160-400)	200 (120-320) vs 233 (160-400)
Required HFNC/NIPPV	32 (23%)	6 (18%)	8 (24%)	9 (26%)	9 (26%)	21% vs 26%	25 % vs 22%
Required ICU/Stepdown	13 (9%)	2 (6%)	2 (6%)	6 (17%)	3 (9%)	6 % vs 13%	12% vs 7%

**Table 4:** Adjusted differences in primary and secondary outcomes for main treatment groups from multivariable regression HFNC: High-flow nasal cannula; NIPPV: non-invasive positive pressure ventilation; ICU: intensive care unit Notes: All statistics presented as a ratio (95% confidence interval); adjusted hazard ratios from the Cox proportional hazard model for time-to-event outcomes; adjusted rate ratios from multiple linear regression with log-transformed outcomes for non-normally distributed secondary outcomes; adjusted odds ratios from multiple logistic regression for binary secondary outcomes; models include adjustment for baseline severity score, diabetes diagnosis, and hospital at home status (H@H) for a patient; analyses for amount of Remdesivir and steroid doses only includes patients who had at least one dose administered during care; length of stay/enrollment defined as number of days from admission/enrollment to discontinuation of care (discharge from hospital or last day of hospital at home); cumulative remdesivir (doses) and steroids (mg) comparisons only using patients who met/required outcome (remdesivir: n = 92, steroids: n = 129).

Outcome		Adjusted Regression Treatment Effects with 95% Confidence Interval			
	Ratio Type	Colchicine vs No Colchicine	p-value	LDN vs No LDN	p-value
Achieved Disease Recovery by Day 5	Odds Ratio	1.76 (0.83 - 3.72)	0.14	1.04 (0.49 - 2.19)	0.92
Day of Disease Recovery	Hazard Ratio	1.32 (0.91, 1.82)	0.16	0.89 (0.63, 1.28)	0.54
Length of Stay, days	Rate Ratio	0.80 (0.59, 1.07)	0.13	0.97 (0.72, 1.30)	0.82
Length of Enrollment, days	Rate Ratio	0.85 (0.67, 1.07)	0.17	1.02 (0.81, 1.29)	0.88
Received Remdesivir	Odds Ratio	0.83 (0.39, 1.79)	0.64	1.30 (0.60, 2.78)	0.50
Remdesivir Amount (doses)	Rate Ratio	0.86 (0.72, 1.02)	0.08	0.92 (0.77, 1.09)	0.32
Received Steroids	Odds Ratio	0.32 (0.05, 2.22)	0.25	2.50 (0.38, 16.39)	0.34
Steroid Amount (mg)	Rate Ratio	0.90 (0.69, 1.17)	0.42	0.84 (0.64, 1.09)	0.19
Required HFNC/NIPPV	Odds Ratio	0.66 (0.28, 1.53)	0.33	1.10 (0.48, 2.52)	0.82
Required ICU/Stepdown	Odds Ratio	0.35 (0.10, 1.27)	0.11	1.52 (0.46, 5.08)	0.49

Patients who received colchicine were observed to have a significantly shorter length of enrollment (LOE) as compared to those who received no colchicine (medians: 3.7 days versus 4.5, p=.04). This did not translate to a statistically significant length of stay (LOS) reduction for these patients (medians: 4.7 days versus 5.7, p=.07). There were no significant differences in other secondary outcomes between treatment groups or study arms (Table 3). LOS, LOE, number of Remdesivir doses, and steroids administered were all log-transformed to meet assumptions for parametric analysis. In adjusted regression, there were no significant treatment effects (Table 4). Exploratory analysis results mirrored those in the main work. There was no association between the day of enrollment and outcomes, ruling out any temporal trend.

Colchicine and LDN were safe and well tolerated in all patients who received at least one dose of study medication. Diarrhea/loose stools/Incontinence was the most common adverse drug reaction, with 12% (n = 17) of patients experiencing it during the study. Of these cases, 7 were in the SOC arm (7/17 = 41%); therefore, these occurrences were not associated with either study drug. Otherwise, adverse drug reactions were infrequent and balanced between treatment groups. Patients who received a study drug were more likely to experience nausea/stomach pain/vomiting compared to those in SOC (pc = 12%, pc+ldn = 15%, pldn = 9%, psoc = 3%). Five patients (4 in the LDN-only arm, 1 in the Colchicine & LDN arm) died during the study period, 2 while still hospitalized and 3 in the weeks following discharge after pursuing comfort care. Study investigators determined that these deaths were not related to study participation.

## Discussion

This study is the first to evaluate the possible reduction in progression to severe disease in patients hospitalized with COVID-19, not requiring higher levels of oxygen support, randomized to receive colchicine, LDN, or both. COLTREXONE did not identify any statistically significant improvement in the unadjusted proportion of patients achieving the primary endpoint of disease recovery by enrollment day 5 within any of the study arms. When the primary endpoint was adjusted for baseline clinical severity score, diabetes diagnosis, and having received H@H services, the non-significant result of the unadjusted analysis was preserved for both colchicine and LDN. 

Secondary outcome analysis did not demonstrate significant treatment effects on remdesivir/corticosteroid use, required HFNC/NIPPV, or ICU admission for any of the study arms. Regression estimates showed a trend toward a prolonged time to recovery with LDN (aHR 0.89; 95% CI: 0.63-1.28, p=0.54) and a trend toward a decrease in the day of disease recovery and LOE with colchicine (aHR 1.32; 95% CI: 0.91-1.82, p=0.16). Although colchicine appeared to reduce the LOE by 0.8 days in the unadjusted data (3.7 days versus 4.5, p=0.04) as well as suggest a clinical trend toward a shorter length of stay (4.7 days versus 5.7, p=0.07) after adjustment colchicine had no significant treatment effect on LOE or LOS. Therefore, this trial did not demonstrate a benefit for either LDN alone or in combination with colchicine. 

Colchicine has been evaluated for patients with COVID-19 infection [15-20]. Since the development of this study, controlled trials involving 1,279 and 11,340 hospitalized patients respectively, COLCOVID [23] and RECOVERY [24], demonstrated that colchicine was not associated with a reduction in 28-day mortality, duration of hospitalization, or the risk of being ventilated. Notably, these studies included patients with higher oxygen support requirements (NIPPV, intubation). A Cochrane Database systematic review [25] through May 2021 found little to no difference in mortality, worsening of clinical status, or improvement of clinical status with colchicine plus standard care compared to standard care alone. A meta-analysis [26] evaluated randomized trials through October 2021 and found no significant difference with colchicine in the odds of mortality but did find a significant reduction in the duration of hospital stay. Both the GRECCO19 trial [16] and the trial by Lopes et al. [17] enrolled hospitalized patients, with similar baseline clinical scores and supplemental oxygen needs as those enrolled in this study. The GRECCO19 trial found a one-day decrease in the duration of hospitalization, while Lopes reported a two-day reduction, noting that both trials used overall higher colchicine dosing than this trial. These findings may be consistent with the nonsignificant trend toward reduced length of enrollment and shorter length of stay found in our patients receiving colchicine which paralleled other non-significant benefits such as reduced remdesivir and glucocorticoid utilization, and fewer patients requiring NIPPV/ICU-level care. Thus, we did not find significant benefit for treatment with colchicine alone in these patients although there was a trend towards non-significant benefit.

The strengths of this study are the careful documentation of changes in SOC throughout the trial. Dedicated teams included pharmacists, and infectious diseases, pulmonology, hospital medicine, cardiology, and rheumatology physicians. Additionally, efforts were made to minimize barriers to study access for diverse patients. 

Limitations of this study include the use of a SOC control in place of placebo given the limited time and resources during the COVID-19 surge. Adapting to emerging treatment recommendations resulted in unknowns in calculating sample size as well as ensuring clinically relevant outcomes, which resulted in adjustments as the study progressed. Additional resources may have increased enrollment capacity to detect a smaller, but still clinically relevant, treatment effect. Also, the incidence of disease recovery for both study drugs was impacted by the rapidly improving SOC for treating patients as the study progressed (remdesivir, glucocorticoids, and tocilizumab). These led to lower progression of disease and better disease recovery in the SOC arm than initially expected. While these effects may have led to a diminished benefit of the study medications, this is outweighed by the benefit of having data reflective of the current SOC and microbiological environment. Lastly, 14% of study subjects received care in the H@H program. Program logistics typically resulted in ≥2 days of H@H care and prevented continuation of study medication outside of the physical hospital. As our study included all H@H days as part of LOE and LOS calculations, patients receiving H@H were not eligible to meet the clinical definition of disease recovery until discharged from the program. As a result, these patients were found to be 81% less likely to meet the clinical definition of a score of 1 by day 5 of study enrollment which may have impacted both our primary endpoint and length of stay data.

## Conclusions

In conclusion, this prospective, randomized, open-label trial in patients hospitalized with COVID-19, not initially requiring high-level oxygen support, did not identify a reduction in progression to severe disease associated with colchicine, LDN, or the combination of both treatments. Non-significant clinical trends toward a potential lower length of enrollment and length of stay were seen in patients treated with colchicine. Further research will likely inform approaches toward other repurposed or novel antiviral agents in the future. 
